# Scale-Free Fluctuations in Behavioral Performance: Delineating Changes in Spontaneous Behavior of Humans with Induced Sleep Deficiency

**DOI:** 10.1371/journal.pone.0107542

**Published:** 2014-09-15

**Authors:** Jeremi K. Ochab, Jacek Tyburczyk, Ewa Beldzik, Dante R. Chialvo, Aleksandra Domagalik, Magdalena Fafrowicz, Ewa Gudowska-Nowak, Tadeusz Marek, Maciej A. Nowak, Halszka Oginska, Jerzy Szwed

**Affiliations:** 1 M. Kac Complex Systems Research Center and M. Smoluchowski Institute of Physics, Jagiellonian University, Kraków, Poland; 2 Department of Cognitive Neuroscience and Neuroergonomics, Jagiellonian University, Kraków, Poland; 3 CONICET, Buenos Aires, Argentina; 4 Neurobiology Department, Małopolska Center of Biotechnology, Jagiellonian University, Kraków, Poland; 5 Biocomplexity Department, Małopolska Center of Biotechnology, Jagiellonian University, Kraków, Poland; Oregon Health & Science University, United States of America

## Abstract

The timing and dynamics of many diverse behaviors of mammals, e.g., patterns of animal foraging or human communication in social networks exhibit complex self-similar properties reproducible over multiple time scales. In this paper, we analyze spontaneous locomotor activity of healthy individuals recorded in two different conditions: during a week of regular sleep and a week of chronic partial sleep deprivation. After separating activity from rest with a pre-defined activity threshold, we have detected distinct statistical features of duration times of these two states. The cumulative distributions of activity periods follow a stretched exponential shape, and remain similar for both control and sleep deprived individuals. In contrast, rest periods, which follow power-law statistics over two orders of magnitude, have significantly distinct distributions for these two groups and the difference emerges already after the first night of shortened sleep. We have found steeper distributions for sleep deprived individuals, which indicates fewer long rest periods and more turbulent behavior. This separation of power-law exponents is the main result of our investigations, and might constitute an objective measure demonstrating the severity of sleep deprivation and the effects of sleep disorders.

## Introduction

### Sleep deprivation

Although good sleep (like nutrition and physical exercise) is considered a basic contributor to human health and well-being, its chronic deprivation seems symptomatic of modern societies. In the last century, the average sleep duration has shortened from approximately nine hours to no more than seven hours in many countries, e.g., USA [1], UK [2], or Japan [3]. Some recent research shows that both total sleep deprivation and chronic sleep reduction may lead to similar effects in terms of physiological, affective and cognitive consequences (e.g., [4], [5]). The most obvious effect of sleep loss is the daytime drowsiness, an underestimated problem concerning operators of transportation and in other “critical-safety” work settings. Apart from drowsiness, sleep deprivation involves impaired immune, endocrine and metabolic functions, and profound neurocognitive deficits. A list of cognitive sleep loss consequences set in a review by Durmer and Dinges [6] includes a variety of symptoms: from slowed reactions, omission and commission errors, and a decline in working memory performance, to deterioration in divergent thinking and increased likelihood of unproductive problem-solving. Cognitive domains are, however, affected diversely by sleep loss, so that sustained attention deteriorates much more than the performance of challenging working memory tasks [4]. Impairments in performance are accompanied by changes in performance self-ratings [5].

Daytime drowsiness is characterized by an urge to sleep, a lack of energy, and decreased ability to complete tasks, often resembling the state of alcohol intoxication. Slowed movements, reduced facial expressions and muscle tone are typical physical symptoms of being sleepy. Thus, the spontaneous locomotor activity may be considered as an index of alertness and sleepiness.

### Scale-free distributions

Power laws are ubiquitous in nature and have been repeatedly detected not only in physics, biology, earth and planetary sciences, but also in economics and finance, demography, epidemiology, and social sciences [7]–[11]. They are typically observed in the vicinity of continuous phase transitions, where the underlying physical processes and fluctuations of measured physical observables exhibit self-similarity at all scales. Scale invariance is therefore commonly considered a signature of “criticality” indicating the complexity of the system and slow decay of spontaneous fluctuations. Of special interest are the scale-free distributions in time, e.g., the waiting-time distributions of light and dark states in quantum dots [12], or dwell-time distributions in closed biological ion channels [13], where the inverse power-laws of state-duration times have been observed and identified with Poisson shot noise in blinking (opening) events. Accumulating evidence also demonstrates [8], [14]–[16] that the dynamics of spontaneous behavior exhibits scale invariance. Time recordings of locomotor activity in rodents and humans have shown that the spatial and temporal distribution pattern of fluctuations appear unchanged regardless of the time scale of observation, thus pointing to the aforementioned universal scaling laws. In particular, recent studies by Sun et al. [17] indicated that the scaling exponent of the power law detected in temporal autocorrelation of activity significantly correlates with the severity of Parkinson's disease symptoms. Similarly, universal scaling laws have been found in locomotor activity periods of humans suffering from major depressive disorders [8]. The disruption of the characteristic universality classes of such laws has been further addressed by Proekt et al. [14] in studies on dynamics of rest and activity fluctuations in light and dark phases of the circadian cycle.

This paper continues along this line of research, seeking to determine standards for measurable criteria by discriminating human behavioral organization as impaired by sleep deficiency. In particular, the aim of the present study is to find whether the same individuals show measurable differences when undertaking two different styles of everyday life. The chosen experimental setup and subsequent statistical analysis allow us to observe such disparities in locomotor activity between subjects sleeping regularly and those undergoing chronic sleep deprivation. As discussed further, those distinct variations in patterns of activity can be already detected in the first day of sleep deficit, leading to significantly different scaling exponents for fluctuations of actigraphy recordings in both groups. When interpreting the findings we also point to their potential application in identifying patients with sleep disorders.

## Materials and Methods

Actigraphy measurements were performed on healthy individuals over one week of their normal life [rested wakefulness (RW)] and one week of partial sleep deprivation (SD) (access to the data: [18]). The circadian cycle of both groups differs substantially: while RW individuals have relatively long “nights” and short “days”, members of the SD group are characterized by a reversed pattern of longer “days” and shorter “nights”, which clearly influences their activity/rest patterns. To overcome this problem we normalized the “days” and “nights” of both groups to the same length, as explained in the **Data analysis** subsection. The resulting time series were statistically analyzed and compared to former studies [8] performed in the “normal” (RW) phase. Since bouts of activity/rest obey different distributions of duration, the best choice of a threshold(s) differentiating between the two states seems to be crucial, and is thoroughly discussed in the **Results** section.

### Participants

Twenty four paid volunteers (12 females and 12 males; mean age 22.7 years, S.D. = 1.6) participated in the study. They were all healthy, non-smokers, and drug-free. They were asked to limit alcohol and caffeine intake during the experimental weeks. They reported regular sleep patterns and no sleep-related problems, controlled with Pittsburgh Sleep Quality Index [19] and Epworth Sleepiness Scale [20]. Participants were informed about the procedure and goals of the study, and provided their written consent. The study was approved by the Bioethics Commission at Jagiellonian University.

Among the 24 sets of data collected 17 were selected for further analysis (8 females and 9 males; mean age 22.8 years, S.D. = 1.8), the other 7 were corrupted by either removing a recording device during the experiment, or not following the sleep schedule.

### Data acquisition

The data acquisition comprised of one week of unrestricted sleep according to individual needs, i.e., rested wakefulness (RW), and one week of daily partial sleep deprivation (SD), with a two-week gap in between the two measurements. Half of the subjects began with the RW phase followed by the SD phase, while the other half had the order reversed. During the sleep deficit week, the participants were asked to shorten their sleep by 33% of their ‘ideal sleep’ by delaying bed-time and using an alarm clock in the morning. The precise length of the restricted sleep was calculated individually for each participant, where the individual sleep need was determined on the basis of the questionnaire administered before entering the experiment (“If you were totally free to plan your day and had no duties at all, at what time would you go to sleep and get up?”). In half of cases the self-reported length of sleep was verified with actigraphy before entering SD phase.

For the 17 selected subjects the average unrestricted sleep, as measured by actigraphy, was 8 h 16 m 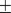 44 m (mean 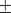 S.D.); in the sleep deprivation conditions it was curtailed by 2 h 20 m 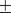 56 m (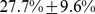) and amounted 5 h 57 m 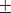 37 m.

Movement tracking was recorded with Micro Motionlogger SleepWatch (Ambulatory Monitoring, Inc., Ardsley, NY), worn on the participant's non-dominant wrist. The data were collected in 1-minute epochs in the Zero-Crossing Method (ZCM) mode, which counts the number of times per epoch that the activity signal level crosses zero (or a threshold very close to zero). The working limitation of the ZCM mode is the difficulty in registering the acceleration of movements, which may potentially cause high frequency artifacts to be counted as a considerable movement.

### Data analysis

#### Day and night or wakefulness and sleep

The raw actigraph data collected in the ZCM mode represent the number of counts per epoch as a function of time. The first step in the analysis is the localization of the ‘day’ 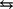 ‘night’ transition (or wakefulness 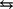 sleep). This is done by the procedure consisting of smoothing the raw data over longer periods of time (usually tens of minutes) and counting actigraph activity above a predefined threshold 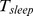. The periods where rest is predominant (above given percentage 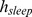) are counted as sleep. If they are interrupted by instantaneous activity, e.g., due to a change of body position, they are glued together. The resulting period is defined as sleep or ‘night’. The remaining time is called wakefulness or ‘day’.

Next, one has to avoid artifacts connected with forced, different lengths of sleep/wakefulness in the two week period under study. Whereas the RW individuals sleep on average 8 hours and are alert 16 hours per day, the SD ones sleep approx. 3 hours less and stay alert 3 hours longer. To make the two samples comparable, out of the total 24 hour period we take 5 hours of sleep, which are common to all participants, and 16 hours of consecutive wakefulness, again common to both groups. We call it the (*5*+*16*) *mode*. For comparison, we also analyze the reversed combination: 16 hours of wakefulness followed by 5 hours of sleep, denoted as (*16*+*5*).

#### Threshold(s) separating states of activity and rest

To precisely distinguish the rest periods from periods of activity, a threshold separating the two states must be chosen. We resolve this problem in two different ways: with a single or double threshold. In the single threshold method we select one specific value of ZCM activity, 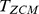, which best separates the two types of behavior investigated. We also test the sensitivity of analyzed distributions to changes of 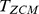 around the optimal value.

The double threshold procedure, in turn, is a hysteresis-like method, in which two step values 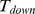 and 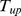 are introduced. We then define the beginning of the activity period as the moment when ZCM activity exceeds 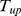, and the end of this period when the activity falls below 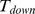. At that moment the state of rest begins, and it terminates when surpassing 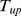. We compare the results following from these two approaches.

#### Statistical analysis

The raw actigraph data 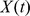, split into activity and rest, are subject to further statistical analysis. We count the number of activity/rest periods of a given duration and calculate the resulting probability density function (PDF) 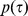 of duration time 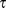. To better assess the statistics of rare events in tails of PDF's we construct, as the main measure of the discussed phenomena, the (complementary) cumulative distribution 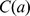 of duration lengths 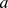:
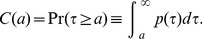
(1)


The function represents the survival probability for the system to stay in a given state up to the time 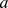. For a stationary time series the survival probability 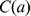 is expected to have a characteristic scale (relaxation time 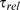) related to the probability per unit time 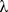 to undergo a change of the state (see Appendix). Put differently, 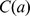 is then a simple exponential function of dwell times, 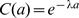, with 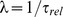.

In order to check the degree of temporal correlations in the activity recordings, the power spectrum 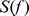 of the signal 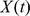 was derived from the Fourier transform of the signal correlation function
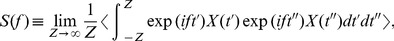
(2)and evaluated for several time periods. The parameter 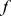 denotes the inverse of time 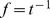.

The numerical estimates of cumulative distributions were fitted with two mathematical formulae: a power-law of the form
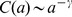
(3)for rest periods and a stretched exponential form
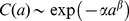
(4)for activity periods. The fitting was performed using log-log or log-linear data, respectively, in order to account for the tails in the distributions. The fitted parameters 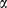, 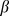, and 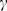 were then compared for several combinations of time periods and subjects.

Each cumulative distribution was constructed from rest/activity periods collected over a single day [i.e., a single (*5*+*16*)- or (*16*+*5*)-hour period] from all the 17 participants in RW or SD condition. The statistics for individual subjects is insufficient to construct reliable cumulative distributions. Consequently, for each day we obtained a single data point characterizing the distribution of the whole group in RW condition and a single point for SD condition.

Throughout the paper the parameters characterizing a single cumulative distribution of rest/activity periods are given together with the standard error of the fit (e.g., exponent 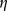 of power spectra); whenever we take an average of the fitted parameters over the whole week, the weighted mean - marked with a horizontal bar - together with the standard deviation is given (e.g., 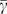). In order to compare RW and SD groups, such means of parameters fitted for several consecutive days were compared with two-tailed Student's 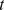-tests performed at 95% confidence level, preceded by a set of tests for equal variances. These tests were performed with Mathematica 9.0 software [21] (functions *TTest* and *VarianceEquivalenceTest*).

Since the cumulative distributions, and consequently the parameters, may change depending on 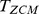 or 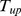 and 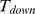, the thresholds were chosen so as to optimize goodness of fit of the two models (3)–(4) and thus best separate rest and activity periods. Whereas fitting the stretched exponential did not show any discernible optimum in the range 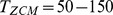, fitting the power law had a clear optimum in several goodness of fit measures (namely, minima in sum of squares error 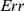, 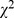 statistic, Akaike and Bayesian Information Criteria). As a result we chose the numerical values of thresholds 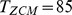 and 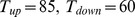 based on this criterion. The details of the fitting procedures and measuring the goodness of fit are described in File S1.

## Results

### Raw data and their spectra

**Figure 1:**
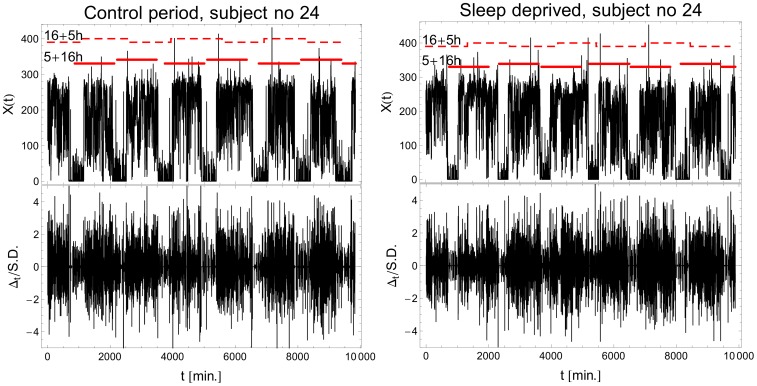
Examples of typical activity recordings. (Left panel) Activity of a control subject (RW mode), and (right panel) of a sleep deprived subject (SD mode). The overall nonzero activity counts 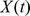 are depicted on the vertical axis. The lower row displays the time series of standardized increments 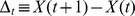.

**Figure 2:**
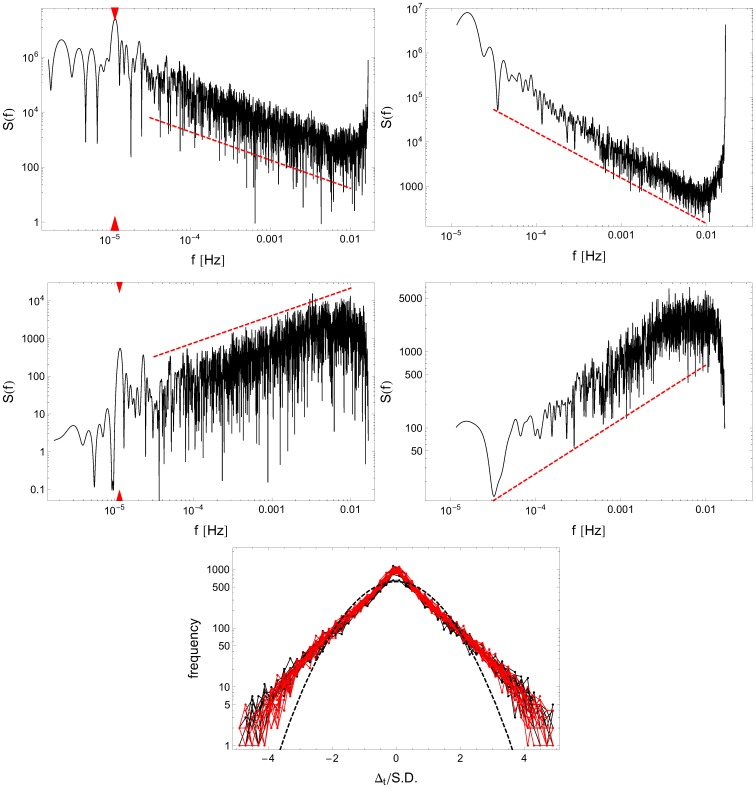
Temporal universality of signal 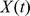 depicted for a typical subject (No. 24). Top panels show the spectral densities 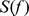 evaluated (left panel) for the one-week experimental time series (i.e., top panels of Fig. 1) and (right panel) for 24 hr periods averaged over the week. In both cases slopes of fitted power laws (dashed lines) exhibit 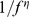 behavior with the characteristic exponent 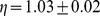 (left panel) and 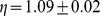 (right panel), respectively. In the middle two panels the similar spectral analysis is shown for the time series of increments (i.e., bottom panels of Fig. 1) exhibiting 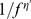 scaling with exponents 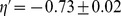 (left panel) and 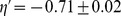 (right panel). These results clearly indicate that the activity events are long-range correlated and the corresponding stochastic process of switching between active/non-active periods is not memoryless (Markovian). The peaks located at 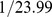 hours, marked by red triangles in the left panels correspond to the circadian rhythm. The bottom panel shows the frequency histogram of standardized increments 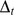/S.D. (black - unrestricted sleep; red - sleep deprivation) compared to a Gaussian probability density function of the same mean and variance (dashed line) on log-linear scale.

The raw actigraph data 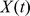 collected in the ZCM mode for an exemplary participant are shown in Fig. 1. The pattern of the activity events in RW mode (top-left panel) exhibits a highly-visible circadian rhythmicity, whereas episodes of activity measured in the SD mode (top-right panel) present a substantial portion of a short-interval chattering. The separation of “days” and “nights” has been performed with 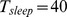 and 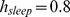. The red solid and dashed lines above the data mark our normalized selection of periods under investigation, (5+16) hours and (16+5) hours, respectively. Additionally, in the bottom panels of Fig. 1 we plot the time series of increments 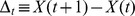 normalized by its standard deviation (S.D.). To assess the nature of fluctuations in 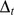, we analyzed distributions of 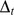/S.D. and the representative frequency histogram (which, by construction, stands for estimation of the probability density function of scaled fluctuations 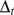/S.D.) is drawn in the bottom panel of Fig. 2 along with a Gaussian probability density function of the same mean and variance. The “experimental PDF” is clearly leptokurtic: pronounced heavy tails show that by comparison to the Gaussian distribution, fewer low-amplitude fluctuations and many more large ones are observed in experimental sets, thus demonstrating their strong deviation from the standard central limit theorem.

The degree of correlation between subsequent intensities of activity 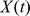 is well represented by its spectral density 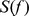. The illustrative plots for a single individual and different time span (one week and 24 hours) are drawn in Fig. 2. For the RW data sets, corresponding to a couple of hundred events, the power spectra exhibit a clear universal, algebraic scaling law 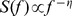, with the scaling exponent 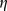 close to 1 [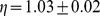 (left panel) and 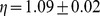 (right panel)]. The respective values for subject 24 in the SD mode are 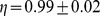 (week) and 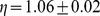 (day). The middle row of the same figure displays the power spectra obtained for increments 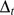 with slopes 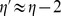 confirming long-range correlations in the fluctuations of intensities 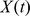.

Consequently, these data demonstrate that the analyzed stochastic process 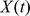 representing variability in intensity of activities is not of the white-noise type, in which case the consecutive events would have been memory-free and the correlation function would have been a Dirac delta function with the corresponding spectral power 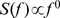.

### Cumulative distributions and their fits; single threshold analysis

**Figure 3:**
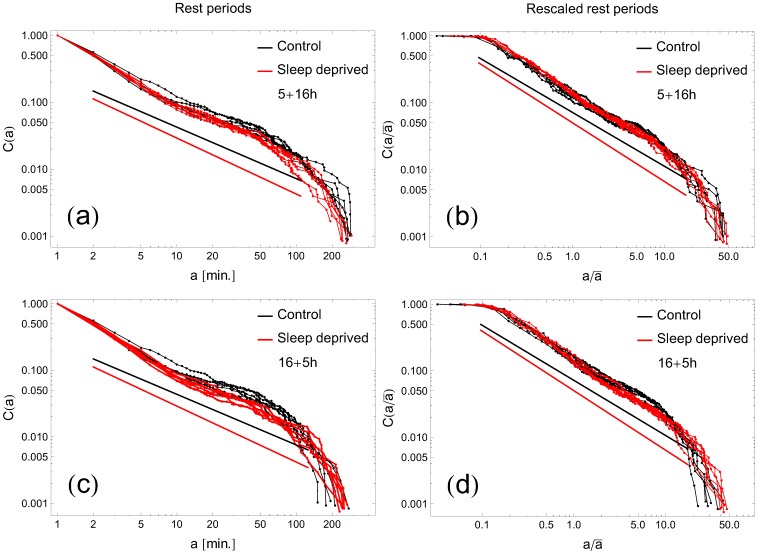
Cumulative distribution of resting times in human motor activity. Double logarithmic plots represent (panels a, c) cumulative distribution 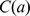 of rest periods as a function of duration time 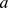 and (panels b, d) cumulative distribution 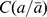 of rescaled rest periods as a function of rescaled duration time 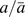 for all RW subjects (black symbols) and all SD (red symbols). Here, 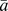 stands for the individual mean of rest period duration. Each curve corresponds to one of six consecutive days. Continuous lines show slopes of the fitted power-laws. Top panels (5+16) mode; bottom panels (16+5)mode. The threshold separating state of activity versus rest 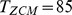.

**Figure 4:**
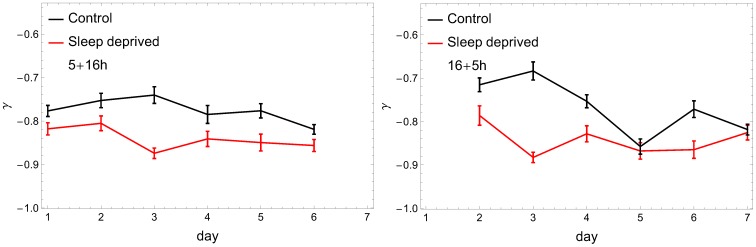
Exponent of the rest-periods distributions for RW (black) and SD subjects (red) as a function of consecutive days. The cumulative distribution assumes (over almost two decades) a power-law form 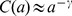. Notation as in Fig. 3. In both cases the characteristic index 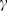 is significantly lower for SD.

With the choice of the threshold 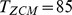, the cumulative distributions 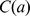 of rest period durations, averaged over all participants, are plotted in Fig. 3a for RW and SD modes in the (5+16) combination. Analogous graphs in the (16+5) combination are depicted in Fig. 3c. Individual curves represent data for consecutive days. To minimize possible influence of individual variations among participants, we have replotted the same cumulative distributions 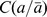 as functions of 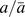, where 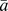 is the individual average, cf. Fig. 3b and d. All curves stay close to straight lines on the log-log scale over 2 order of magnitude in time, which is a signature of power-law behavior of the form given by Eq.(3). We performed fits of the cumulative distributions for each curve and present the extracted exponent 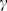 in Fig. 4. There are several conclusions which can be inferred from this analysis. Firstly, we do not see significant differences between the (5+16) and (16+5) recordings. Secondly, the 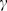 values of the control group are close to those reported earlier [16] for healthy humans (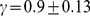 versus average 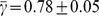 in this study).

**Figure 5:**
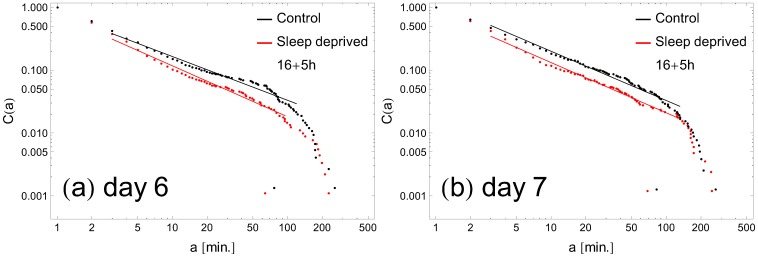
Sample distributions of rest periods with fitted slopes. The continuous lines are best fits; their extent indicates the range of data points fitted (the first and last fifteen data points are not fitted). Panel (a) is an example where both the distributions and the fitted slopes can easily be distinguished. Panel (b) shows an example in which the fitted slopes are nearly equal, although the difference of distributions is clearly visible.

Finally – and we consider this observation our main result – the analysis performed indicates a significant difference in behavioral motifs between the control group and sleep deprived individuals. The higher coefficient 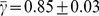 derived for sleep-deficient individuals emphasizes the fact that the pattern of their resting times consists of more short periods and, respectively, fewer longer inactivity time intervals than in the control group. This observation contrasts with the results for the rest-time distributions of depressed humans [8], [16], where lower scaling exponent 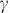, and thus heavier tails in the cumulative distribution, were observed for disordered individuals. The difference in the exponent 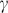 that we observe is significant: a two-tailed Student's 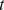-test between sets of resulting 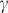 coefficients for the RW and SD groups was performed at 95% confidence level (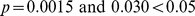 for (5+16) and (16+5) settings, respectively) providing evidence for a statistically notable difference between means of these two groups. It is interesting that after the change of the scaling parameter 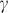, already on the first day of sleep deprivation (Fig. 4), we do not observe any additional trends during the subsequent days. One should also notice that even if the exponents are close to each other on some days (e.g., days 5 and 7 in Fig. 4 right panel), the distributions are clearly distinguishable from each other, as can be seen in Fig. 5, where 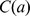 in the rest state for SD and RW individuals are plotted for exemplary days 6 and 7.

**Figure 6:**
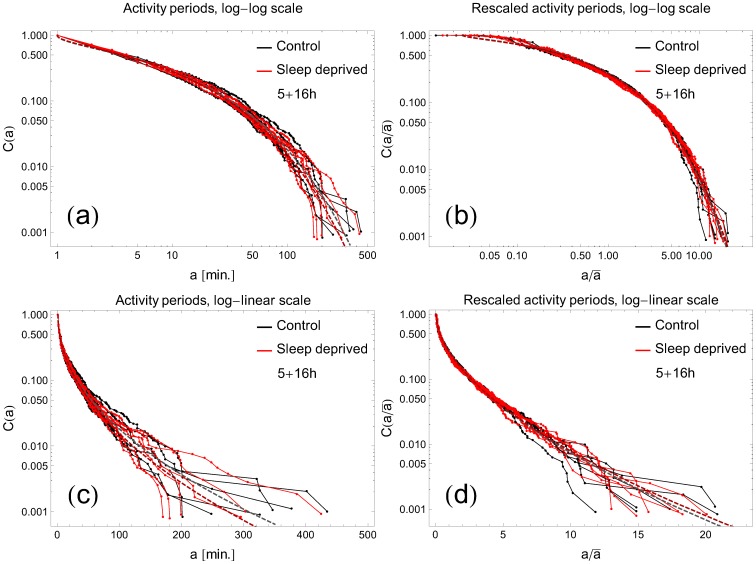
Cumulative distributions of activity periods. The curves on log-log and log-linear scales follow the typical pattern for a stretched exponential 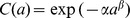 as a function of (panels a, c) the length of activity periods 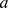 or (panels b, d) the length 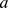 rescaled by the individual average 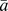. All curves collapse on a similar stretched exponential function; there is no significant difference between RW and SD subjects. The threshold determining rest and activity period has been preset to 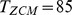.

In an analogous manner, the cumulative distributions of the activity periods for RW and SD cases are plotted as functions of 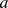 on log-log and log-lin scales in Fig. 6a,c; furthermore, Fig. 6b,d presents those distributions as functions of 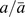. Both original and rescaled cumulative distributions of activity periods collapse well onto the stretched exponential form Eq.(4). The values of the fitted parameters 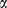 and 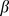 for RW and SD groups are not significantly different (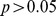, two-tailed t-test). This result remains in agreement with previous studies on humans and rodents [8], [15], [16].

**Figure 7:**
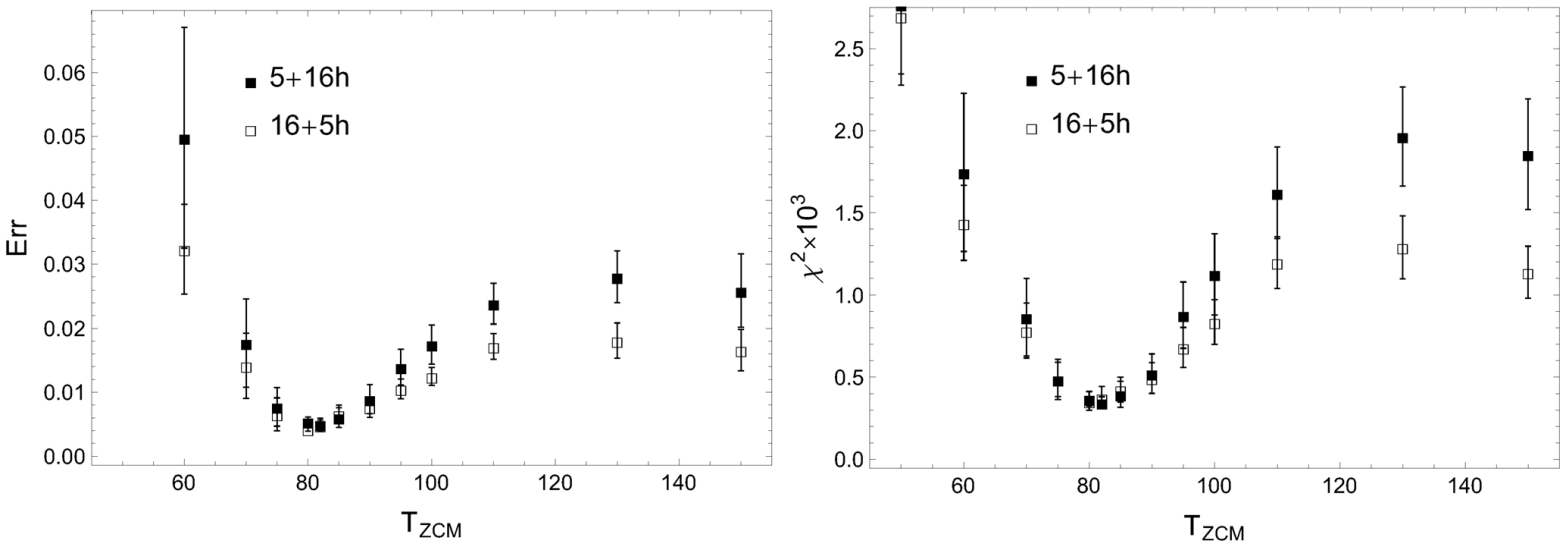
Dependence of goodness of power-law fit on the threshold 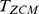. The data were collected for unrescaled rest periods of RW subjects. The residual sum of squares (left), the reduced 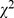 statistic (right) and other measures of goodness of fit (see File S1) show a broad minimum in the same range 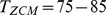, which serves as a criterion for choosing 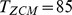 for further analysis.

The choice of thresholds separating the activity and rest periods is an important issue. By selecting one particular value of ZCM activity we separate two different temporal distribution profiles: the power law for states of rest and (stretched) exponential for states of activity. It is thus clear that there must be an optimal value that best distinguishes the two. The criterion we used to find it was the best fit of the resting-state cumulative distribution 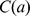 to the power-law form. The resulting sum of squares error 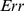, along with the goodness of fit expressed by 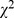, are plotted in Fig. 7 as functions of the threshold value 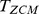.

### Robustness of the results: Cumulative distributions and their fits in double threshold analysis

**Figure 8:**
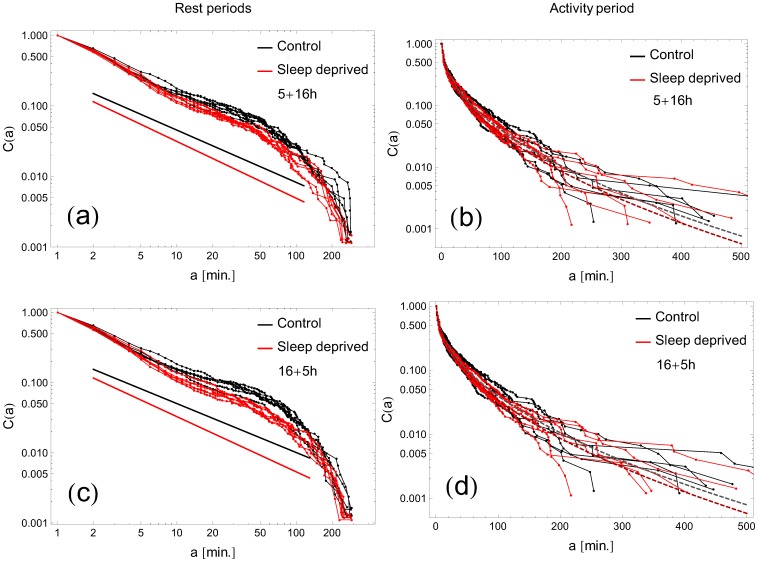
Cumulative distributions of rest and activity periods with hysteresis-like thresholding. Two-threshold analysis of rest and activity periods with 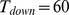 and 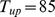. Notation as in Figs. 3 and 6.

**Figure 9:**
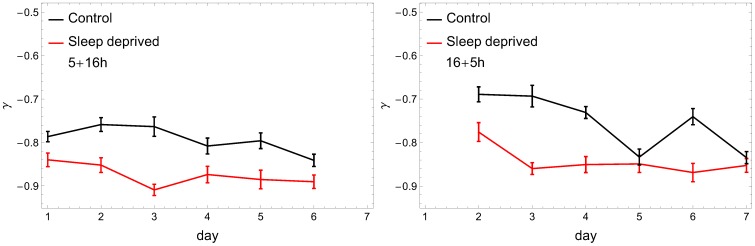
Exponents of the rest-periods distributions with hysteresis-like thresholding. Thresholds chosen as in Fig. 8. Notation as in Fig. 4.

All the results discussed above were obtained with the single threshold definition (Fig. 1–7). In order to further test the sensitivity of the results to the definition of a threshold, we refined our analysis by considering a hysteresis-like distinction of the locomotor activity (see section **Data analysis**). With the choice 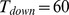 and 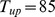 we plot the cumulative distributions 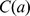 of rest (Fig. 8a,c) and activity (Fig. 8b,d) time intervals for both RW and SD groups, together with the fitted exponents of the power-law cumulative distribution of resting states (Fig. 9). In the case of activity periods no clear difference between the RW and SD individuals was observed. The overall fit (averaged over days and individuals) yields for the RW sample 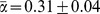 and 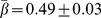. Similar values for the SD mode are not significantly different (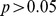; two-tailed t-test). In contrast, the difference between the control and sleep deprived individuals can be well observed in the profiles of the rest-periods cumulative distributions. Numerically, the power-law fit yields 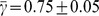 for RW and 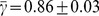 for SD samples. Altogether, we observe that cumulative distributions obtained with this definition agree with those obtained in the single thresholding method, therefore confirming the robustness of the results obtained.

## Discussion

The evidence of motion recorded by actigraphs is broadly used in medical practice aimed at understanding typical circadian-rhythm patterns of healthy subjects, and at detecting possible rhythm disorders in humans. Although the method is not indicated for the routine diagnosis, the actigraphy recordings may be useful in quantitative evaluation of the variety of neuropsychiatric diseases. Specific analytical methods applied to actigraphy records can reflect various features of physiological activities and serve as an outcome measure in characterizing disturbances of the circadian-rhythm patterns and alterations in locomotor activity in specific populations like children (apnea testing) or individuals suffering from depression [16], Parkinson's disease [17], schizophrenia [22], or dementia [23].

In the former studies by Nakamura et al. [8], [16], actigraphy was employed to determine the difference in human behavioral organization between healthy subjects and patients with major depressive disorders. The authors analyzed activity and resting time durations and found that the cumulative distributions follow a stretched exponential form for activity periods and scale-free, power-law behavior for resting time intervals. Moreover, by analyzing the behavioral organization of humans suffering from major depressive disorders, they found significantly lower power-law scaling exponents for the rest-period durations than for healthy control groups.

In analogy to those investigations, we have performed the experiment and applied statistical evaluation of the results, keeping in mind that our RW and their healthy control groups should reach similar conclusions. Therefore, e.g., the choice of activity threshold which separates state of activity from rest has been validated by the use of a criterion similar to [8], [16], i.e., the overall average of nonzero activity counts.

Evaluation of residence-time distributions and calculation of a mean waiting time to an activity event are broadly studied problems in biological physics and have been addressed in a number of studies [9], [13], [14], [24]–[29]. Stochastic systems with internal states in which discrete events (like neuron firing or exceeding a threshold of activation) occur at a state dependent rate frequently exhibit long-time persistent correlations, which are well reflected in power spectra of the representative signal recordings [13], [15], [30]. At the same time, many natural phenomena, including spontaneous human behavior, demonstrate non-homogeneous Poisson or even mixed, non-Poisson distributions of events, with intensity rates identified with scale-invariant Lévy statistics [31].

Lévy-stable distributions are a class of self-similar infinitely-divisible probability laws [32], [33] and by virtue of the Lévy-Khinchin theorem are represented by distributions which can be uniquely decomposed into two independent – Gaussian and Poisson – superposition parts. This structure is further responsible for the heavy-tailed amplitudal surges in the composition of Lévy fluctuations and for long-ranged temporal dependencies characterized by the slow decay of their autocorrelation function. With this observation in mind, we have discussed the possible emergence of a scale-invariant survival probability 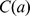 in a stochastic process of switching between two different activity states of the system (cf. Appendix) pointing out that the two derived probability laws are related to each other through asymptotic properties of individual relaxation modes.

Higher values of the exponent 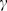 for sleep-deprived subjects signals less heavy tails of waiting-time distributions in an immobile (resting) state than in an analogous distribution for the control group, and can be associated with restlessness/inquietude and increased variability (burstiness) of activity in recorded time series. Consequently, such alteration of locomotor behavior can be a representative sign of disorders related to sleep-deficiency and possibly, in line with results presented in former studies [8], [16], [17], [34], a valuable diagnostic fingerprint discriminating between healthy and depressed/disordered individuals.

It seems likely that some aspects of both the waking period (length of prior wake, local use of neuronal networks) and the sleep period (sleep length and continuity) may explain performance deficits and the restoration of function. Accordingly, deeper theoretical understanding of different forms of sleep loss would be possible by comparing them together, in a single experiment [35].

Finally, it is important to recognize that the analysis of activity patterns of chronically sleep deprived subjects will not necessarily reveal their decreased capabilities. Compensatory efforts and recruitment of additional brain resources could intervene to make the activity level appear unaffected. It could be that unraveling these disturbances requires more sophisticated analysis, or that the subtle neurocognitive effects of sleep loss can be observed only with the use of refined neuroimaging techniques, possibilities that deserve to be explored in future work.

## Appendix: Dynamics of Activation and Omnipresence of Power Laws – Theoretical Considerations

We have studied the statistics of resting (inactivity) times and confirmed that the derived distributions are of power-law type 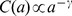 with the index 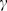 taking a fixed value over two orders of magnitude in time. In turn, activity period durations for control and sleep-deprived subjects obey a stretched exponential form for a wide range of recordings. We claim that both behaviors can be understood as different, asymptotic facets of an anomalous relaxation law governed by the generalized Mittag-Leffler probability distribution function describing rates of individual processes underlying switching from states of rest to activity.

The pattern of time-duration (the overall pattern of distribution of fragments of time spent in a state of a given activity) is related to the rate function of a point process featuring events following spontaneous discharge (escape) from the inactivity (or alert) state. The spectrum of times between subsequent events can be described mathematically by a probability density function (PDF) 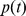 (which is equivalent to the experimentally derived frequency distribution of periods). For very small 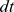, the probability that the event of escape from the state will happen in time range 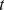 to 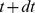, given no such event occurred by times prior to 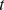, can be expressed in terms of the conditional probability 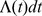, where 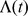 denotes the instantaneous “rate” at which the sequence of subsequent “ticks” of representative events occurs in time. By introducing the probability that “the system remains in the state intact up to the time 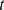
”, otherwise called the survival function 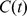


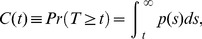
(5)the conditional probability 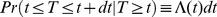 can be rewritten as
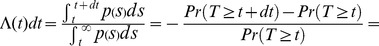


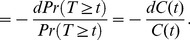
(6)


This leads to a simple relation between the rate function 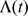, the survival probability 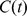, and the PDF 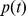, which takes the form of
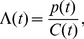
(7)with the differential equation for the probability that no change of state occurs at least until time 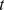:
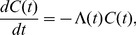
(8)whose solution depends on the relevant choice of 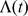:
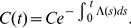
(9)


Note that cumulative probability that a state change happens by time 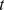 can be expressed as
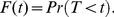
(10)


Accordingly, for 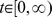 we will have 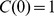 [i.e., 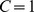 in Eq.(9)], 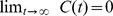 and 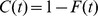. From the above analysis it also follows that
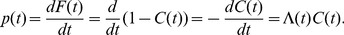
(11)


In particular, for a uniform rate 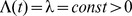, one gets
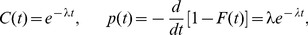
(12)which is typical for a homogeneous Poisson point process, where the probability 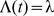 is assumed constant throughout the whole time interval of interest. The mean duration time spent in a given state (say, before a discharge from the state takes place) is then given by 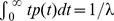. If, on the other hand, the time dependence of the rate function 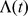 is assumed to scale differently with small and large 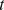 as, e.g.:
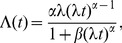
(13)which for 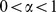, 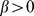 leads to
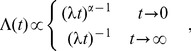
(14)the survival probability is given by
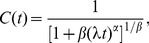
(15)corresponding to the frequency distribution of time durations
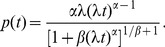
(16)


The above formula Eq.(16) represents the probability density of the Burr distribution exhibiting two power-law behaviors
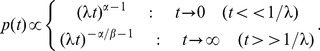
(17)


The survival function Eq.(15) possesses interesting limiting properties: As 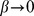, 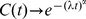, that is, it tends to the stretched exponential form (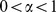) with the characteristic power-law behavior of the corresponding probability density for 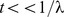:
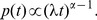
(18)


Note that as 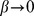 and 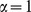, the common, exponential form of the survival function 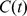 is recovered. In turn, for 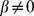 and 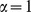, the 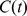 function tends to
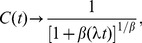
(19)which yields a tail of the Pareto distribution in the expression for the probability density distribution 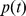:
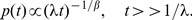
(20)


The non-exponential form Eq.(15) and, consequently, the time dependent character of the rate 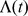 may follow from a statistical probability mixing, e.g., from randomization of the rate 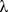 (cf. Eq.(12)).

To clarify this point, let us rewrite the survival function as a conditional probability
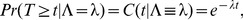
(21)where 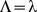 denotes random rate 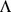 taking value 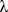. By taking an average with respect to PDF 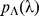, the final 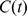 takes the form
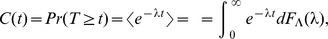
i.e., it is given by the Laplace transform of the rate distribution function. In this case the “effective” rate is derived from
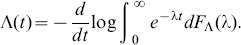
(22)


In fact, it is easy to check that, if the rate PDF takes the Dirac-delta form
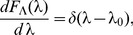
(23)the relation Eq.(22) yields a constant function 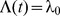 and the corresponding PDF of duration times spent in a given state has an exponential form Eq.(12) with 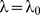. On the other hand, it can be shown that the rate Eq.(22) takes the form Eq.(14) if the intensity distribution function 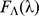 is of the generalized Mittag-Leffler form:
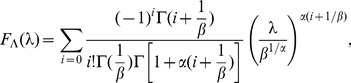
(24)which itself represents a mixture of the completely asymmetric Lévy-stable 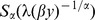 and the gamma 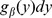 distributions
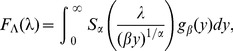
(25)where
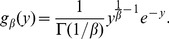
(26)


Altogether, the above analysis classifies stretched exponential and Pareto distributions as probability laws stemming from the same origin, i.e.: either from the randomization of an individual rate 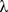, or correspondingly, from the randomization of relaxation time 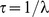, the parameters which describe dynamics of stochastic activation events. The interpolation property of the Mittag-Leffler function (stretched-exponential character for short times with a transition to long-time inverse power-law behavior) is an appealing feature, and has been observed experimentally in various realms like protein conformation dynamics [36], dielectric relaxation in complex media [26], [30], [37] or financial market time series [38]. It well may be that the same function can serve in modeling temporal turnover effects in neurological recordings.

## Supporting Information

File S1 **Details on data processing and fitting.** The file includes analysis of goodness of fit of cumulative distributions for one and two activity thresholds; it contains 5 figures.
(PDF)
