# Supplementary material for: Scale-Free Fluctuations in Behavioral Performance: Delineating Changes in Spontaneous Behavior of Humans with Induced Sleep Deficiency
Source: PLoS One. 2014 Sep 15;9(9):e107542. doi: 10.1371/journal.pone.0107542 (PMC4164638; doi:10.1371/journal.pone.0107542)
Supplement: File S1 — Details on data processing and fitting. The file includes analysis of goodness of fit of cumulative distributions for one and two activity thresholds; it contains 5 figures. (PDF) [file pone.0107542.s001.pdf]

**Supplementary Information to *Scale-free fluctuations in behavioral performance: Delineating changes in spontaneous behavior of humans with induced sleep deficiency* by Jeremi K. Ochab et al.**

# 1 Preparation of the data

The locomotor activity data were acquired with 1 minute resolution. For the unrescaled data, the probability density distribution was estimated by performing 1 minute binning; for rescaled data, where the durations were divided by a subject's average, the minimal rescaled period duration was taken (which varied between $0.015 - 0.1$). The complementary cumulative distribution function (CDF) was then calculated with the same binning. To allow better fitting, the step-like behavior of the cumulative distribution had to be avoided, and so, for each plateau only the rightmost point was retained. (This procedure may be understood in terms of varying bin widths, so that only the steplike *changes* in the distribution are recorded.)

# 2 Fitting the data

The fitting of the rest-period CDF was performed on logarithmically transformed data (the logarithm of both the period length and the probability was taken). A linear least-squares fit was then performed with unitary weights for all data points

$$\ln[C(a)] = -\gamma \ln(a) - c.$$

This means that the tails of the distribution were taken into account with the same weights as the rest of the distribution; if one does not take the logarithm of CDF values, the tails lie very close to zero, and consequently squares of residuals in that region are always small and do not have much influence on finding the optimal fit. The first two points of the CDF and the last 15 were excluded from the fitting (as shown, e.g., in Fig. 5), so that only the power-law regime was fitted.

The fitting of the activity-period CDF was performed on logarithmically transformed probabilities only (i.e., as shown in Fig. 6c,d); a nonlinear least-squares fit was performed with unitary weights for all data points, as before.

$$\ln(C(a)) = -\alpha a^{\beta}.$$

# 3 Goodness of fit: measures

We utilize six measures of goodness of fit:

1. the sum of squares error for the logarithmic data

$$Err_{log} = \sum_{i=1}^{N} (\ln(C_{data}(i)) - \ln(C_{fit}(i)))^2$$

2. the reduced chi-square statistic for the logarithmic data

$$\chi^2_{log} = \frac{1}{dof} \sum_{i=1}^{N} (\ln(C_{data}(i)) - \ln(C_{fit}(i)))^2 \,/\, (\ln(C_{data}(i)) + \ln(C_{fit}(i)))$$

3. the sum of squares error

$$Err = \sum_{i=1}^{N} (C_{data}(i) - C_{fit}(i))^2$$

4. the reduced chi-square statistic

$$\chi^2 = \frac{1}{dof} \sum_{i=1}^{N} (C_{data}(i) - C_{fit}(i))^2 \,/\, (C_{data}(i) + C_{fit}(i))$$

5. Akaike information criterion

$$AIC = 2k - 2L$$

6. Bayesian information criterion

$$BIC = k \ln(N) - 2L,$$

where $dof$ stands for the number of degrees of freedom, $N$ is the number of data points (i.e., the number of bins of the cumulative distribution), $k$ is the number of parameters in the model fitted, $L$ is the likelihood function, and $C_{data}(i), C_{fit}(i)$ are the value of the complementary cumulative distribution in the bin $i$ and the predicted response value, respectively.

Since the data we fitted were logarithmic, the measure minimized by the fitting algorithm was the residual sum of squares $Err_{log}$ (1). This is a standard measure used for comparing different models fitted to the same data set. This quantity, nevertheless, does not seem to be a correct indicator of goodness of fit if one compares fits for different sets of data, which differ in the number of data points and have systematically higher or lower values. The reduced (i.e., divided by degrees of freedom) $\chi^2_{log}$ statistic takes both of these issues into account.

In Fig. S1, for comparison with other works we also give sum of squares and chi-square (3)-(4) for non-logarithmic data (although the fits were kept logarithmic, as above). We also report on AIC and BIC (5)-(6). All of these, however, provide similar information, and we do not use them later on.

The values shown in Figures S1-S4 are averaged over the entire week (days 1-6 for 5+16hr setting and days 2-7 for 16+5hr).

# 4 Optimal fit: dependence on one threshold

We checked threshold values between $50 - 150$, for both RW and SD, activity and rest periods, original and rescaled period durations, and 5+16hr and 16+5hr settings. The goodness of stretched exponential fits of the activity periods' data does not exhibit any single distinguishable minimum. As there is also no significant difference in activity periods between RW and SD, we disregard it in the choice of the optimal threshold value.

The rest periods on the other hand show a clear minimum of goodness of fit in all instances. For rest periods of RW with non-rescaled period durations, in Fig. S1 we show all the measures (1)-(6); since they contain the same information, for the remaining data (Fig. S2-S4) we show only reduced $\chi^2$ plots.

For further analysis, we chose the threshold value $T_{ZCM} = 85$, as it lies within the optimal region. The analysis in this paper, however, was also performed for other thresholds with the same main result that the exponents $\bar{\gamma}$ for RW and SD are significantly different. This can be seen in Fig. S3.

It is also noteworthy that for the optimal choice of the threshold the difference between the two settings: 5+16hr and 16+5hr disappears. The choice of the threshold does in general influence the resulting CDF exponent $\bar{\gamma}$. Coincidentally, its value also has a broad minimum in a similar threshold range (Fig. S3).

# 5 Optimal fit: dependence on two-thresholds

In Fig. S4, two minima of $\chi^2_{log}$ can be seen: one in the range of $T_{up} = 80 - 110$ for $T_{down} = 30 - 100$, and the other in the range $T_{up} > 120$ for $T_{down} = 5 - 20$.

For low $T_{down}$ and high $T_{up}$ the power-law fit is indeed very good, with a clear cut-off, as can be seen in Fig. S5 (red). However, when $T_{up} > 200$, the threshold is to high and the activity and rest periods begin to mix. Increasing the threshold also entails a decrease in the number of data points and an increase in the values of CDF, as illustrated in Fig. S5.

In summary, the goodness of fit of two-threshold minima (see $\chi^2_{log}$ in Fig. S4) is comparable with one-threshold minimum (see $\chi^2_{log}$ in Fig. S1)

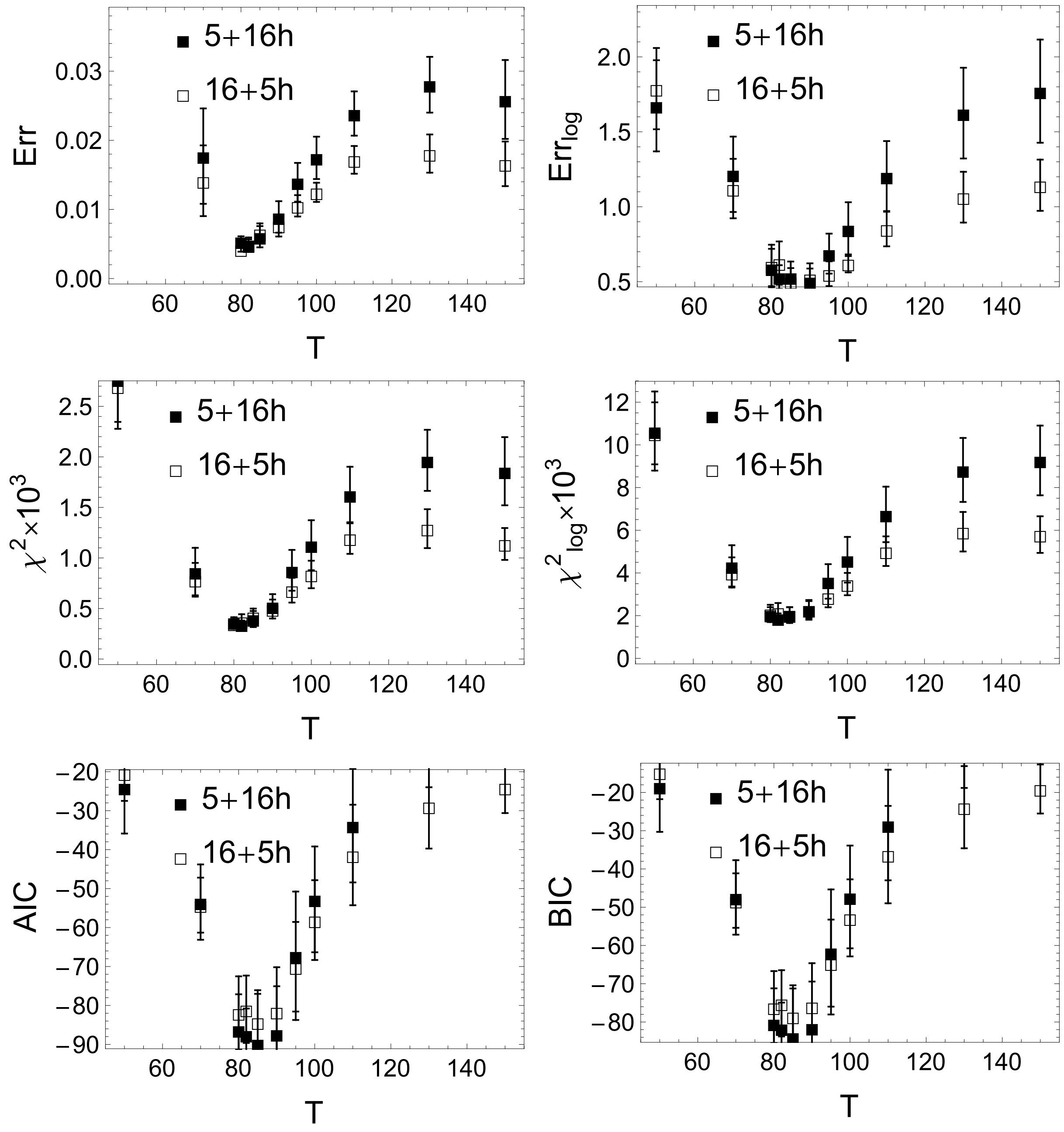


Figure S1: **Goodness of fit for rest-period distribution depending on activity threshold** $T$ **(RW).** A linear model was fitted to log-log cumulative distribution data; the fit minimized $Err_{log}$ sum of squares. Each data point is an average of the goodness measure over six days. Error bars represent standard deviations.

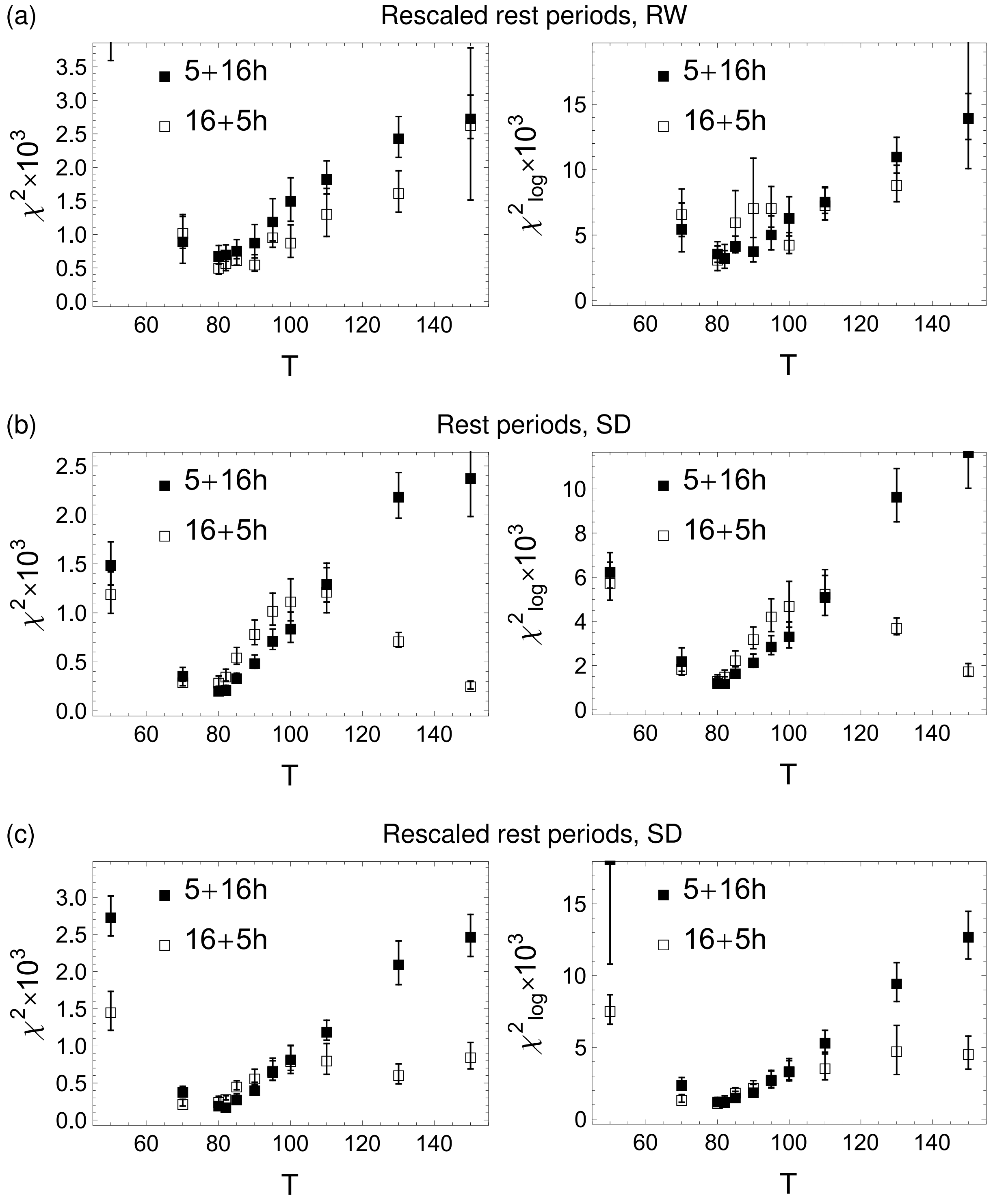


Figure S2: **Goodness of fit for rest-period distribution depending on activity threshold $T$.** Error bars represent standard deviations.

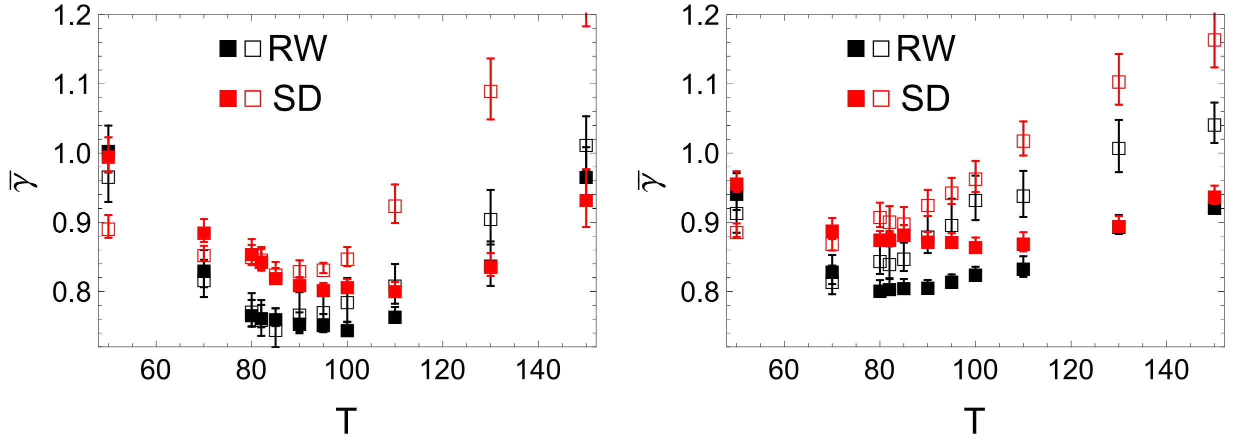


Figure S3: **Fit parameter $\bar{\gamma}$ (average over entire week) for rest distribution depending on activity threshold T.** Closed symbols correspond to 5+16hr scheme, while open to 16+5hr; black and red to RW and SD modes, respectively. (Left) original and (right) rescaled period durations. The exponent $\bar{\gamma}$ consistently differs between RW and SD in the range $T = 70 - 110$. Error bars represent standard deviations.

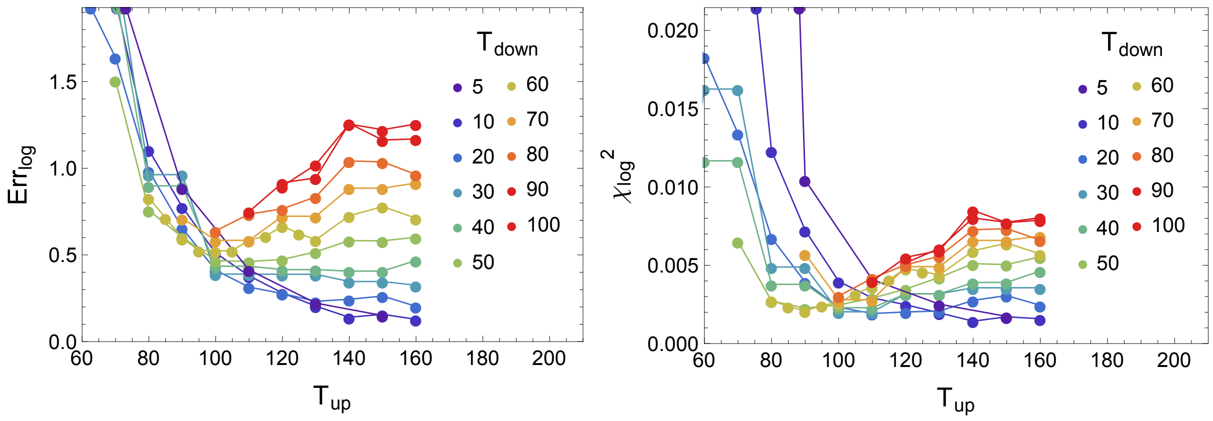


Figure S4: **Goodness of fit for rest-periods distribution depending on the activity thresholds $T_{up}$ and $T_{down}$.** A linear model was fitted to log-log cumulative distribution data; the fit minimized $Err_{log}$ sum of squares. Each data point is an average over six days in RW mode, 5+16hrs setting. For clarity, the standard deviations of data points are not shown.

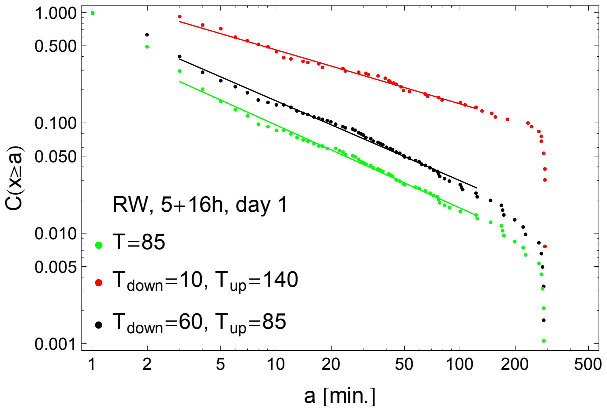


Figure S5: **Example of cumulative distributions for different optimal thresholds.** The $\chi^2_{log}$ values are respectively: $0.0014(5)$ for $T = 85$ (green), $0.0016(4)$ for $T_{down} = 10, T_{up} = 140$ (red), $0.0019(8)$ for $T_{down} = 60, T_{up} = 85$ (black), with $67, 71$, and $50$ data points respectively.
